# Novel Modeling of Task vs. Rest Brain State Predictability Using a Dynamic Time Warping Spectrum: Comparisons and Contrasts with Other Standard Measures of Brain Dynamics

**DOI:** 10.3389/fncom.2016.00046

**Published:** 2016-05-12

**Authors:** Martin Dinov, Romy Lorenz, Gregory Scott, David J. Sharp, Erik D. Fagerholm, Robert Leech

**Affiliations:** Computational, Cognitive and Clinical Neuroimaging Laboratory, Division of Brain Sciences, Department of Medicine, Imperial College LondonLondon, UK

**Keywords:** DTW, DFT, oscillations, EEG, fMRI, EEG-fMRI, microstate analysis, predictability

## Abstract

Dynamic time warping, or DTW, is a powerful and domain-general sequence alignment method for computing a similarity measure. Such dynamic programming-based techniques like DTW are now the backbone and driver of most bioinformatics methods and discoveries. In neuroscience it has had far less use, though this has begun to change. We wanted to explore new ways of applying DTW, not simply as a measure with which to cluster or compare similarity between features but in a conceptually different way. We have used DTW to provide a more interpretable spectral description of the data, compared to standard approaches such as the Fourier and related transforms. The DTW approach and standard discrete Fourier transform (DFT) are assessed against benchmark measures of neural dynamics. These include EEG microstates, EEG avalanches, and the sum squared error (SSE) from a multilayer perceptron (MLP) prediction of the EEG time series, and simultaneously acquired FMRI BOLD signal. We explored the relationships between these variables of interest in an EEG-FMRI dataset acquired during a standard cognitive task, which allowed us to explore how DTW differentially performs in different task settings. We found that despite strong correlations between DTW and DFT-spectra, DTW was a better predictor for almost every measure of brain dynamics. Using these DTW measures, we show that predictability is almost always higher in task than in rest states, which is consistent to other theoretical and empirical findings, providing additional evidence for the utility of the DTW approach.

## Introduction

Dynamic Time Warping (DTW) has been extensively used in data mining, but also in pattern recognition and classification. It is not an overstatement to say that it is today one of the central techniques in the data mining community (Keogh and Kasetty, 2002; Ding et al., 2008a; Rakthanmanon et al., 2012). DTW is a dynamic programming (DP) based technique for finding the best alignment between two time series/data sequences. It can take into account phase shifts and other non-linear changes in the time series, unlike the much simpler (but computationally faster) Euclidean distance based alignments (Rakthanmanon et al., 2012). While typically used in a univariate setting, it has also been successfully used in multivariate contexts as well (Bankó and Abonyi, 2012; Górecki and Łuczak, 2015). While very similar methods also based on DP have revolutionized and are core in other biological and scientific fields, especially in bioinformatics in the form of BLAST, FASTA, and other sequence alignment methods (Smith and Waterman, 1981; Gotoh, 1982; Edgar, 2010; Di Tommaso et al., 2011), neuroscience has not yet explored this powerful technique nearly as much. In fact, sequence alignment is behind most of genetic, proteinogenic, phylogenetic and other molecular and genetic biology work and results. In recent years it has been picked up in conjunction with other machine learning or statistical methods for various neuroimaging and neuroscientific investigations, including improved ballistocardiogram artifact detection and removal (compared to using a template or average based artifact removal method) (Niennattrakul and Ratanamahatana, 2007; Kustra et al., 2008; Annam et al., 2011), decoding of speech from intracranial electrode recordings (Zhang et al., 2012), modeling and decoding spectrotemporal feature differences for overt and covert speech from cortical recordings, (Martin et al., 2014), better discriminating ERP latency differences (Zoumpoulaki et al., 2015), to distinguish movement-related to stimulus-related activity (Perez et al., 2013) and modeling dynamic task-based functional connectivity in an EEG task (Karamzadeh et al., 2013), to name some. We hoped to explore new uses of the technique, applying it to simultaneously recorded EEG-fMRI data set, to find how it may be useful in capturing oscillatory properties of the data (for the EEG data), and how it might compare or stand next to other data analysis approaches on the same data sets, which relevant to our interests in the relationships between neural dynamics and oscillations, criticality, EEG microstates and fMRI networks. In particular, we were interested in how well DTW can be used to find how much oscillatory activity (e.g., sinusoidal) there is at specific frequencies, as opposed to the discrete fourier transform's (DFT) non-specificity for oscillatory contribution to the derived spectrum—since DFT has no choice but to give you activity at a given frequency *even if there is no specific oscillation or activity at that frequency*, due to the nature of the sinusoidal (sine and cosine) basis functions used in FFT-like methods. By running what we call DTW-spectrum (see Methods Section), we find a more interpretable alternative to a DFT-spectrum that correlates strongly with the DFT but seems to capture somewhat different dynamics (which are in fact more predictive of the SSE as well as a few of the other measures). We believe this to be relevant as many papers and researchers make claims of band power being “oscillatory activity” (Klimesch et al., 1997; Kelly, 2006; Osipova et al., 2006; Klimesch et al., 2007; Meltzer et al., 2007). While the two may be highly correlated in most cases and there is causality in one direction (higher amplitude and more frequently observed oscillations lead to greater band power in the respective frequency band) the reverse direction is not causal.

**Figure 1:**
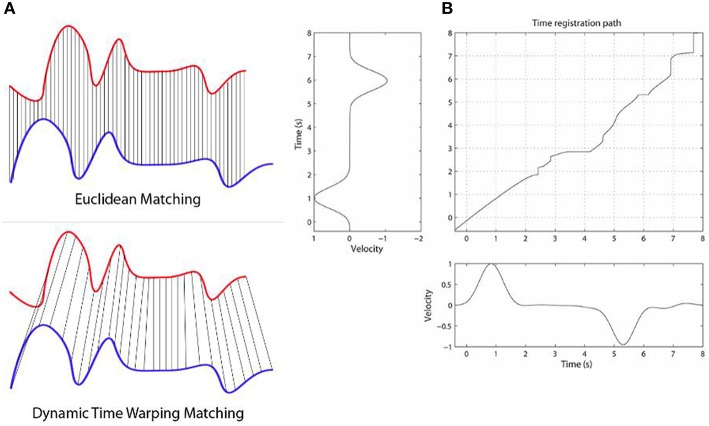
**The Figure illustrates how the DTW method works**. Panel **(A)** shows two time series, that are highly similar, but the blue one is lagging behind the blue one (has a negative phase relative to the blue one). While ED would give a fairly high alignment cost here, DTW will find a fairly low cost, as they are highly similar when the phase shift is accounted for. Panel **(B)** shows a DP matrix for two similar but slightly varying signals. The (possibly non-unique) optimal alignment is found by finding the minimum cost path from the top right corner to the bottom left, after the DP matrix is calculated. Note that while no warping window is displayed here, we can place a diagonal below and above the main diagonal in the matrix as constraints for the warping (e.g., Sakoe-Chiba band). This not only speeds up the alignment (often by a factor of 10 or more), but tends to lead to more accurate alignment results as well. We used such a constraint band for these two reasons. Panel **(B)** is taken from Wong et al. (2015).

We give here a very brief outline of the general DTW method, which is the backbone of the techniques we explore in this work. DTW is a highly flexible DP-based method for comparing the dissimilarity (equivalently, the similarity) between two signals. The flexibility comes from the fact that any discretized signals can be used, as well as very long sequences. The use of dynamic programming here means that the DTW values in the 2D matrix can be defined and computed recursively and fairly efficiently [O(n^2^) in the worst case, though in practice it is much faster]. By caching of previously computed results in the matrix in this DP-way, in practice this leads to efficient quasi-linear or amortized linear (Salvador and Chan, 2007; Ding et al., 2008b) to quadratic (asymptotically) time algorithms for solving every matrix cell. Once the matrix cells are filled, the top right corner contains the overall DTW distance between the two time series, and the minimum alignment or warp path is found by starting at that top right corner and greedily going left, down, or left-down diagonally, until the bottom left corner of the matrix is reached—i.e., at each step taking the lowest possible cost. The warp path is not guaranteed to be unique. The warp path contains all details of the warping process, including phase shifts, stretching and squeezing of the time series, relative to each other, as inferred by the DTW algorithm. Figure 1 below (taken from Rakthanmanon et al., 2012) illustrates how the method works, with two similar but non-identical time series that are mostly just phase-shifted versions relative to each other.

We chose to use DTW as a similarity measure on which to base a more interpretable spectrum calculation as it is among the best distance/similarity measures in existence. It is fast with an amortized linear time running cost (Ding et al., 2008b). Ding et al. (2008b) point out in their thorough investigation of various similarity measures on different data set sizes and types that: “there is no evidence of any distance measure that is systematically better than DTW in general. Furthermore, there is at best very scant evidence that there is any distance measure that is systematically better than DTW in on particular problems (say, just ECG data, or just noisy data).”

In order to test how well the DTW-based methods work for neuroscientific questions, we assessed them against benchmark measures of neural dynamics (that we and others have been using to study a range of neuroscience problems). The DTW-spectrum that we compute gives us a DFT-like spectrum that may be more directly related to the oscillatory/sinusoidal nature of the signal than the DFT spectrum, which can give high values in certain frequency bins even if there is no oscillatory/sinusoidal activity at that frequency. Given that neural dynamics change substantially with cognitive state, we applied the DTW approaches in two different cognitive states (a active, externally task-focused state and a more passive, “resting” state)—we found differences in the predictability of data in the two cognitive states that fits with previous results and with our hypothesis of higher predictability and more ordered neural dynamics during a task-focused state.

We compared the DTW measures (and a more typical DFT approach) to our benchmark measures of neural dynamics including: the predictability of the EEG signal (SSE from a MLP), EEG microstates, EEG cascades (or “avalanches”), as well as simultaneously acquired FMRI BOLD signal. The predictability of neural signals is an important aspect of neural dynamics and varies with cognitive state—therefore, we sought to more directly quantify this by using a multi-layer perceptron (MLP) to come up with a measure of prediction error. Our working hypothesis was that prediction error, as quantified by the sum squared error (SSE) would be better predicted by the DTW than DFT and that this would be a stronger effect in task than at rest (van den Heuvel et al., 2008; Deco and Jirsa, 2012; Hellyer et al., 2014; Fagerholm et al., 2015). In a similar vein, we also sought to investigate neuronal cascades, which have been used to characterize dynamical regimes such as self-organized criticality (Kinouchi and Copelli, 2006; Shew et al., 2009; Deco and Jirsa, 2012; Fagerholm et al., 2015). We used EEG microstates as microstates have been shown to be powerful and simple multivariate approach to looking at EEG data. Microstate duration or the specific microstate just prior to a trial during tasks correlates with EEG alpha band power, fMRI BOLD network properties and activity, ERP characteristics, behavioral measures (e.g., reaction time and miss/accuracy rate), as well as neuropathological conditions such as Alzheimer's or Schizophrenia (Lehmann, 1989; Lehmann et al., 1994, 2005; Fingelkurts, 2006; Jann et al., 2009; Britz et al., 2010; Musso et al., 2010; Van de Ville et al., 2010), depending on the exact microstate (type) or length. They have been called the “atoms of thought” [as EEG microstates seem to reflect both rest and task-dependent neural dynamics on longer timescales (tens to hundreds of milliseconds)] (Michel et al., 2009), reflecting the discrete nature of cognitive processing and current state-dependent response to external events. Studying the relationship between the EEG and FMRI/BOLD is an increasingly active area of research, therefore, we also sought to see how well DTW (and the other measures) would relate to simultaneously acquired BOLD.

## Methods

**Figure 2:**
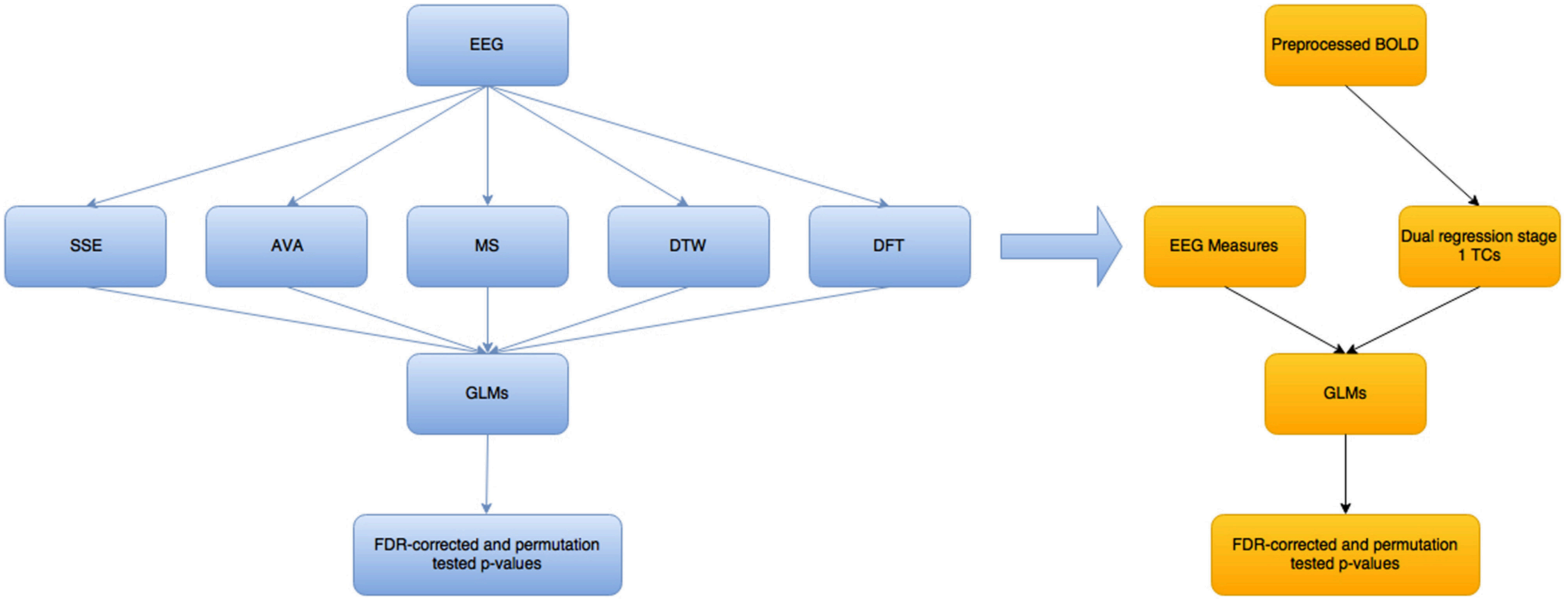
**Overview of the steps taken in this modeling/analysis work**. The blue colored boxes show measures/results related to the EEG part of the analysis, while the yellow-orange part is for the fMRI BOLD part. The EEG measures are not all single measures—there is one SSE and one AVA measure, as well as DTW and DFT, but 2 MS-related measures. These measures are fed as they are into separate GLMs, to try to predict each of the 5 main variables (excluding the mean GFP variable that is related to the MS), using each of the others. The EEG measures were convolved with a standard double-gamma hrf before being fed into the BOLD-GLM to try to predict the dual regression stage 1 time courses (Beckmann et al., 2009).

Figure 2 below shows a high level overview of the methods used and how they relate to each other.

**Figure 3:**
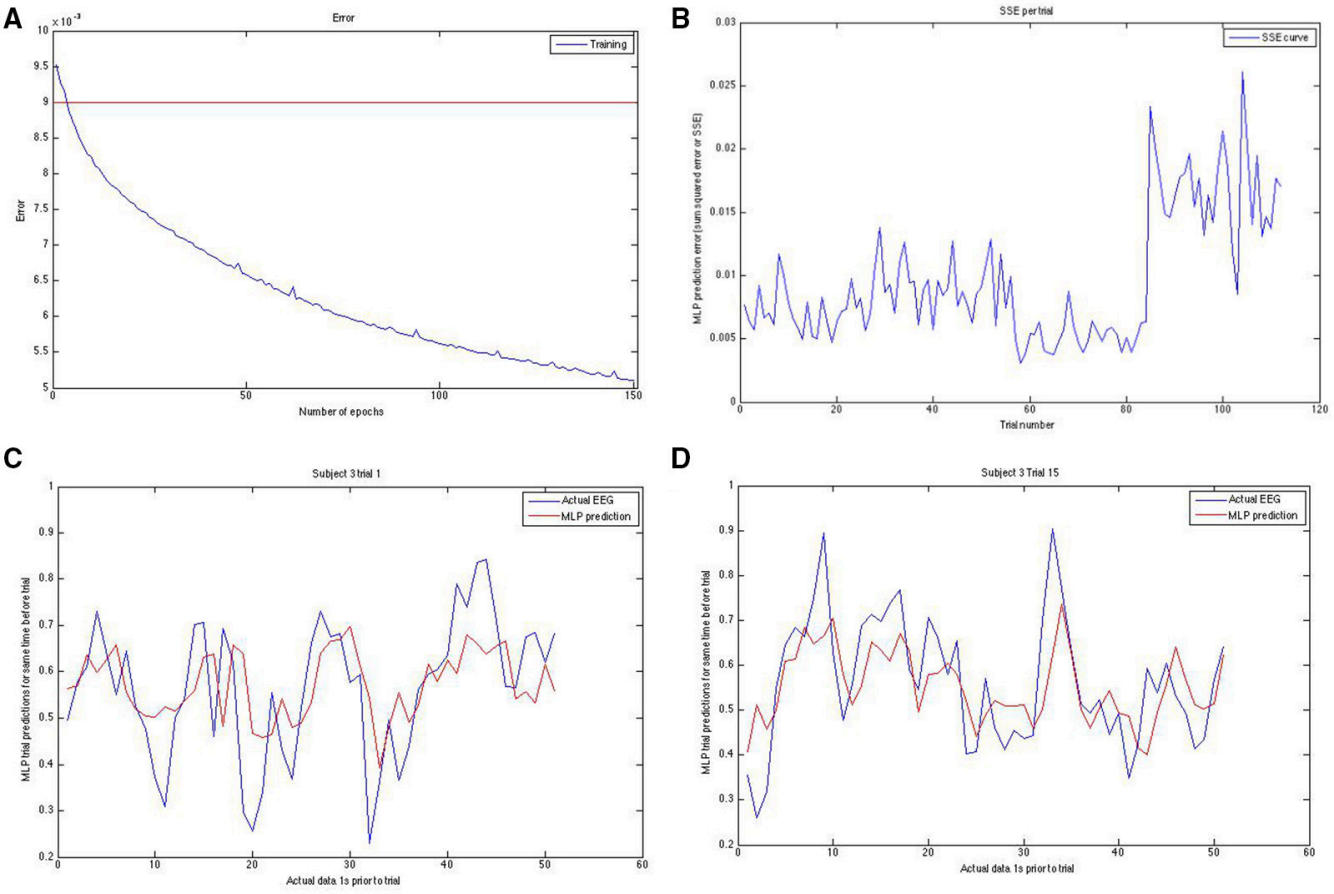
**Panel (A; top left) shows the training error curve for the MLP**. We used only networks that went below a certain training set SSE. Panel **(B)** (top right) shows the SSE on the y-axis plotted against the trial number on the x-axis. Note the erratic sudden spike in SSE in the last block for this run of the modeling on this subject. To reduce the effect of hard-to-predict variable model-dependent effects that could arise, we averaged our results across 10 runs for task and rest predictions both. Panels **(C,D)** (bottom left and bottom right, respectively) show the actual EEG signal (down sampled from raw) in blue and the prediction of it in red for two different trials of two different subjects. Note that prediction follows the general trend well.

The whole preprocessing, processing and analysis pipeline is depicted graphically in Figures 2, 3. Each step is described in more detail in its respective section below, but we outline the pipeline here briefly. We took EEG data from a simultaneous EEG-fMRI study, for which we had corresponding fMRI BOLD data recorded simultaneously. This is a dataset that has been used and published in previous work of ours (Fagerholm et al., 2015). The data involves a Choice Reaction Time (CRT) task with 5 alternating task and rest blocks from 15 subjects. For the task portion of this modeling and analysis study, we used the first task block for training the neural network (described in detail below) and the rest for testing (roughly 80–20 split for training and testing). For the rest blocks, we used the second block for training (as it was slightly longer than the other rest blocks and was therefore different to them), and the rest of them for testing.

To summarize the methods briefly: we computed the DTW-spectrum between 8 and 13 Hz, out of which we took the standard deviation of it to condense the vector into a scalar—one scalar value for each trial, so that we can more directly compare this value with the SSE, DFT-spectrum's standard deviation, and other trial-wise values. We took the standard deviation for both the DTW and DFT in only the alpha band range (i.e., 8–13 Hz). Where we refer to DTW, we mean the standard deviation of the DTW computed for the alpha band of the pre-trial data, and where we refer to DFT, we really mean the standard deviation of the DFT as well, unless specifically stated otherwise. We also computed 2 EEG microstate measures: the microstate length just prior to the trial (i.e., the length of the microstate at trial time). We also computed the mean global field power (GFP) of the microstate for the trial, as well as EEG avalanche/cascade length for the trial/just prior to the trial. We computed the mean GFP as the GFP is a measure of the spatial standard deviation of electrical scalp potential and we thought this could be another meaningful measure alongside the microstate length prior to the trial.

We ran the whole pipeline and modeling 20 times, averaging the relevant results from each run, mainly to minimize randomization-related noise due to model training. We note that we validated the results by running a few 20-repetition averages according to the entire pipeline here, but, unless otherwise noted, report only details and results from a single representative 20-rep average run of the pipeline and modeling.

### Preprocessing

#### EEG data preprocessing

The starting EEG data was the same data used in Fagerholm et al. (2015). MRI induced and amplified artifacts (gradient switching, RF flip, cardioballistic) were removed using the BrainVision Analyzer 2 software from Brain Products GmbH as described in our previous paper. So that we remove some reference-specific bias we re-referenced the data to an average reference. Next, because there were some strong artifacts remaining in the data that highly affected training and predictions with the MLP as well as the computation of EEG microstates, we ran an additional cleaning step using the Artifact Subspace Reconstruction (ASR) method (Mullen et al., 2013), which removes and reconstructs sections of data that are deemed “bad.” The *clean_rawdata* tool that implements ASR in EEGLAB also removes channels deemed too noisy during ASR. Finally, to speed up and improve training and prediction we down sampled the resulting ASR-cleaned data to a sample rate of 50 Hz to speed up training and analysis.

### Measures and analysis

#### MLP EEG prediction

We used a multilayer perceptron (MLP) to predict EEG time courses because the MLP model is relatively simple to implement, train, and test while still being a powerful non-linear model. We note that other statistical approaches could also be applied (and may be more successful), e.g., auto-regressive models. However, we are not interested in optimizing predictive accuracy, *per se*, but rather the relative associations to different measures (e.g., DTW and DFT) and different cognitive states.

**Figure 4:**
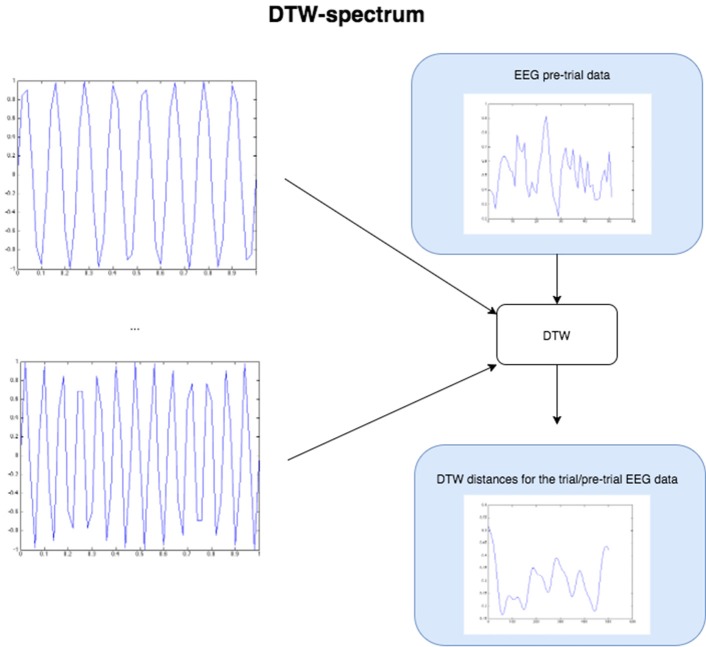
**The Figure demonstrates the computation of the DTW-spectrum**. We generate progressively higher frequency sine waves in a loop, and for each one run DTW of the EEG pre-trial data against that particular sine wave. The resulting distance measure is the distance/dissimilarity between the EEG pre-trial data for that trial and that frequency. If we plot the distance/dissimilarity measures derived for each sine frequency comparison, we get a plot like the one on the bottom-right, which shows what we call the DTW-spectrum. It is almost a horizontal reflection of a DFT-spectrum but appears to capture slightly different (more specific) dynamics. For the statistical GLM modeling, we took the standard deviation of the DTW-spectrum and DFT-spectrum to condense each spectrum to a single value for each trial.

The neural net is a single hidden layer multi layered perceptron (MLP) as implemented by the Rasmus MATLAB toolbox (Palm, 2012) using tanh-based activations, no regularization, a single hidden layer of the same size as the input layer and trained using gradient descent in batch mode (batch size = 50). In order to improve prediction, we fed a block of points to the MLP (40 lagged points, for a total of 41 data points—the time point just before the time point to be predicted, plus the 40 points prior to that one). The MLP training ends up placing greater weighting on more recent time points and less on ones from further in the past as a result of the training, but the lagged points increase the prediction accuracy. We took the MLP prediction errors, as quantified by the SSE for each trial, and tried to predict these trial-wise SSEs using the standard deviation of the DFT power per trial (from here on referred to simply as “DFT”), the standard deviation of the DTW-spectrum (from here on referred to only as “DTW”) and EEG-based avalanches/cascades as well as EEG microstate-derived measures of the trial-preceding microstate length and trial-preceding mean GFP power. We show an example of how we initially confirmed whether the training was working in the Figure 4. We set a fairly strict threshold for the training error and re-trained a new neural net if the training error was above this training set error threshold. We also visually confirmed (as shown in Figure 3) that the predicted and the actual EEG signals match well enough.

We used ~1 min long blocks of task or rest data to train and test a multi-layered (1 or 2 hidden layers) MLP for predicting the next time point separately in task and rest blocks (i.e., different models were trained for task and for rest). We trained a new model for each individual for each model run. The MLP was trained by being provided a block of points (ultimately 50 points) prior to each trial, from multiple channels simultaneously (ultimately 1 channel was used for the group-level results presented, as the prediction becomes noisier with more included channels), to predict up to n points in the future. However, though we could predict significantly well a few points ahead even with multiple channels with 40 lagged points, we used only 1 channel to keep the prediction errors lower and cleaner and as the focus is not on predicting the EEG time series as far into the future as possible but to look at periods of predictability (although we note that it also predicts above change with time points beyond *t* + 1, with worsening error performance). A single channel's prediction and dynamics was deemed sufficient for this.

#### Training

The training data consisted of the following: for each trial within the task blocks we used data 1 s prior to the trial. Starting from trial time—1 s to the trial point, the points were used to predict the next time point right after the points used to predict the predicted point. For rest blocks, there was still a “trial point” recorded and used for convenience, though it was a rest trial and no stimulus was actually shown to the subjects.

#### Model repetition

The MLP parameter space (number of hidden layers, number of nodes per layer, L2 regularization penalty, etc.) was determined manually in a pilot phase on a subset of the data, prior to applying to the full data in the automated pipeline. To prevent local-minima adversely affecting the results, we applied a testing-set error threshold to the training error during the backprop training. If the error was greater than the threshold, we retrained a new model and repeated this until a sub-threshold model was found. Our focus was not to validate the generalizability of the MLP; therefore, we did not perform full model cross validation but did repeat the modeling and GLMs each 10 times, averaging the resulting beta coefficients where appropriate.

We re-ran the whole pipeline multiple times with a number of different parameter choices. Presented are representative results from an averaging of 20 runs, averaging across results to minimize noise due to randomization steps inherent in the MLP training as well as the k-means clustering used for the microstates.

#### DTW measures

Next we describe exactly the DTW-based measure that were computed and used.

**Figure 5:**
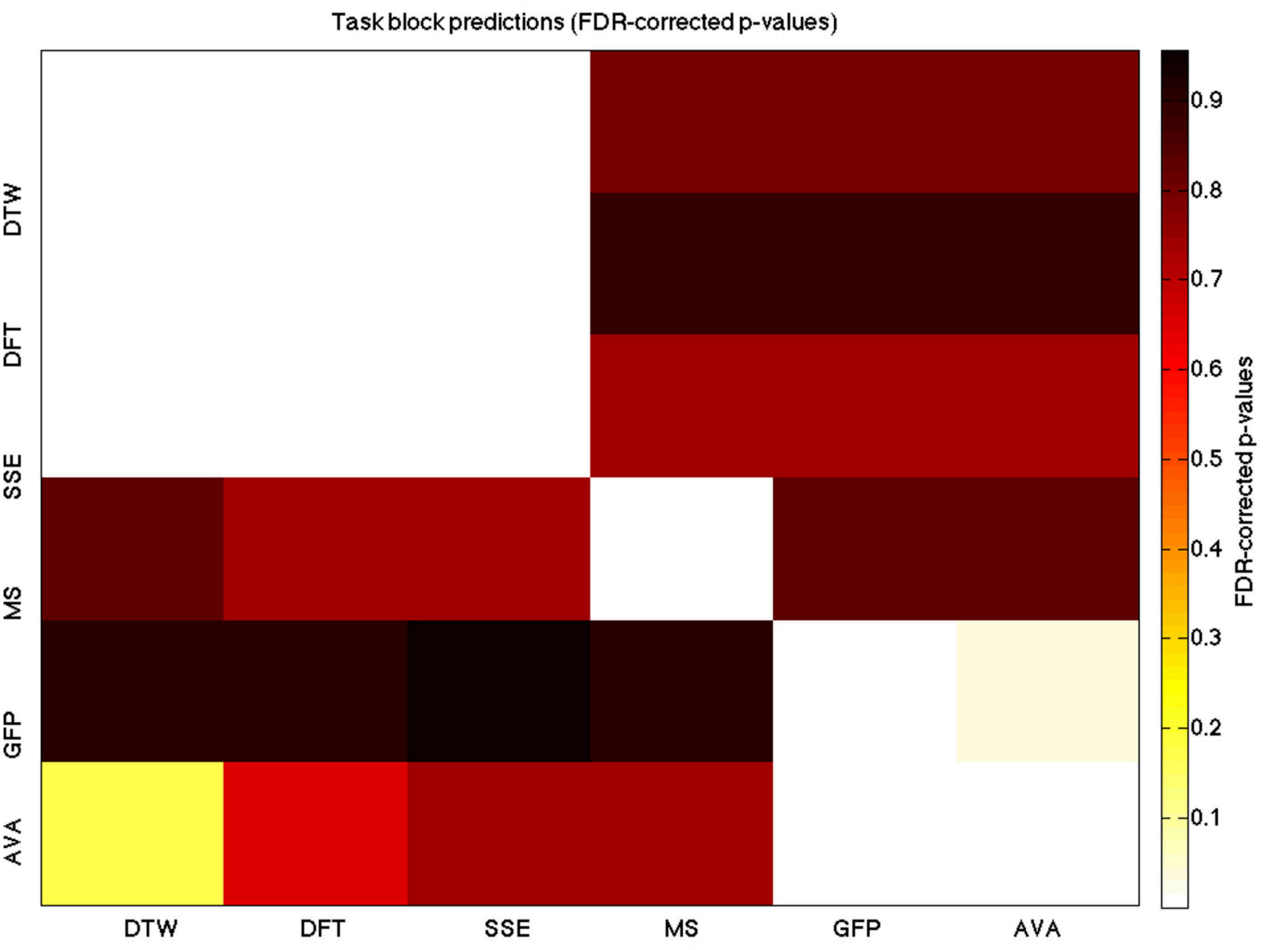
**A heatmap showing FDR-corrected group-level *p*-values from the *t*-tests on the GLM-derived beta coefficients of the task blocks**. The hotter the color (i.e., the closer to white) the closer the value is to 0. The diagonals are all set to 0, for clarity.

The DTW-spectrum is computed as illustrated and described in Figure 5. In short, it is a direct computation of how similar the signal is to sines at different frequencies. Unsurprisingly, this resulted in a spectrum that highly (inversely) correlated with a DFT spectrum computed as usual. In other words, the stronger the value at a given frequency bin of the DFT/FFT, the smaller the value of the DTW-sine value at that frequency bin (since the DTW-sine is measuring dissimilarity). Though the DFT/FFT measures similarity and the DTW-sine dissimilarity, they are expected to provide highly inversely correlated results (just mutually inverted spectra). However, the two methods do not produce identical results. The DFT/FFT is not and cannot guarantee that high values at a given frequency can be interpreted as suggesting high amounts of activity at that frequency. In contrast, the DTW-sine spectrum is more directly interpretable due to the different nature of the method, where there is a direct similarity comparison between a sine wave at a given frequency and a small stretch of the (EEG) signal.

To compute a truer spectrum (compared to DFT's output) of the data, we run DTW against sine waves in a range of frequencies, of the same length as the data that is fed in, with linear step size for the frequency change. This gives us a distance measure of the pre-trial data against each sine frequency. Figure 4 above below illustrates this approach. The result from this algorithm is a vector of DTW distances for each sine frequency, with the resulting values and plot being the DTW-spectrum. It is strongly inverse correlated to DFT, but not identical (see Figure 5 and results). We also tried sawtooth waves at matching frequencies but the results were not nearly as predictive or clear as with sines and all further results are discussed only in reference to DTW-sine-spectrum. We tried a range of warping window sizes but ultimately used a warping window of size 20 for DTW-spectrum calculation. In order to reduce the DFT-spectrum to a single value for each trial (to be compared to and useable alongside the other measures), we computed the standard deviation of the spectrum for use in the statistical modeling (GLMs) in the next part. This of course removes a lot of potentially interesting details but makes modeling easier and still retains some of the relevant dynamics. Ideally, we would have done more sophisticated modeling taking the exact spectra and entire distributions into account.

#### Microstates

Microstates were computed in a standard way (Michel et al., 2009; Van de Ville et al., 2010) by first computing the Global Field Potential (GFP) across all channels (post extra cleaning steps that we applied to the EEG), followed by GFP max peak detection, followed by clustering of these max peak positions (for which we used K-means with *n* = 12). We fed into the K-means the EEG data at the peaks of the GFP, as this is where the maximal signal-to-noise ratio tends to be (Van de Ville et al., 2010). We then had a labeled GFP-peak time course for each individual. We took the scalp maps (EEG values at all electrodes) at each of these GFP-max positions, concatenated across all subjects to form a group-level map set, and did K-means clustering on this to determine the most consistent maps on a group-level (*n* = 12 maps). Once these 12 maps were found, we then went back to each subject and compared each time point of the GFP/EEG time course with these 12 maps, assigning at each EEG time point the map that was closest to the EEG topography at that point. From this we extracted the microstate immediately preceding a trial (whether in rest or task blocks). We then counted how many times points (or the length) of this microstate immediately prior to the trial. We also looked at the mean GFP power of this microstate prior to the trial, as a measure of the mean spatial standard deviation during/just prior to the trial. Both of these measures are used in every GLM.

#### Model averaging

For both task and rest blocks, we collected all measures across the 20 repetitions of the model creation and prediction, averaging the results of those. We then used these 20-rep-averaged model values in the GLMs.

#### Avalanche/cascade

Avalanches, or cascades, were computed as described in detail in Meisel et al. (2013) and Fagerholm et al. (2015). In brief, the z-transformed channel data is thresholded at a standard deviation of 3.2, 3.5, or 3.7, depending on the number of avalanches detected using the point process based detection and a bin width of 2. We selected these SD thresholds in order to have the number of avalanches be roughly equal to the number of trials, to avoid losing a great deal of information when subsequently down sampling to fully match to the number of trials (*n* = 112).

#### fMRI data preprocessing

The same fMRI preprocessed data was used as in Fagerholm et al. (2015). We ran this preprocessed data through stage 1 of FSL's dual regression (Beckmann et al., 2009) using the Smith IC20 ICA maps (Smith et al., 2009). These ICA maps are spatial maps representing statistically related signals across brain regions (as imaged and recorded using fMRI) during rest, which are known to be relevant (appear to active or deactivate—or generally correlate) for both task and resting cognitive conditions. These are commonly used fMRI maps. The FSL toolkit's dual regression stage 1 then extracts subject-specific time courses for each of these fMRI ICA spatial maps. This allows us to look at subject-specific correlations between activity (in time/trials) in these spatial maps and other variables of interest (in time/trials).

## Results

We compared the DTW measures (and more typical DFT approaches) to our benchmark measures of neural dynamics including: the predictability of the EEG signal (SSE from a MLP); EEG microstates, EEG cascades (or “avalanches”), as well as simultaneously acquired FMRI BOLD signal. Below, we examine each of these relationships in turn comparing both DTW and DFT with the benchmark measures in the two cognitive states (as well as compare the benchmark measures with each other).

**Figure 6:**
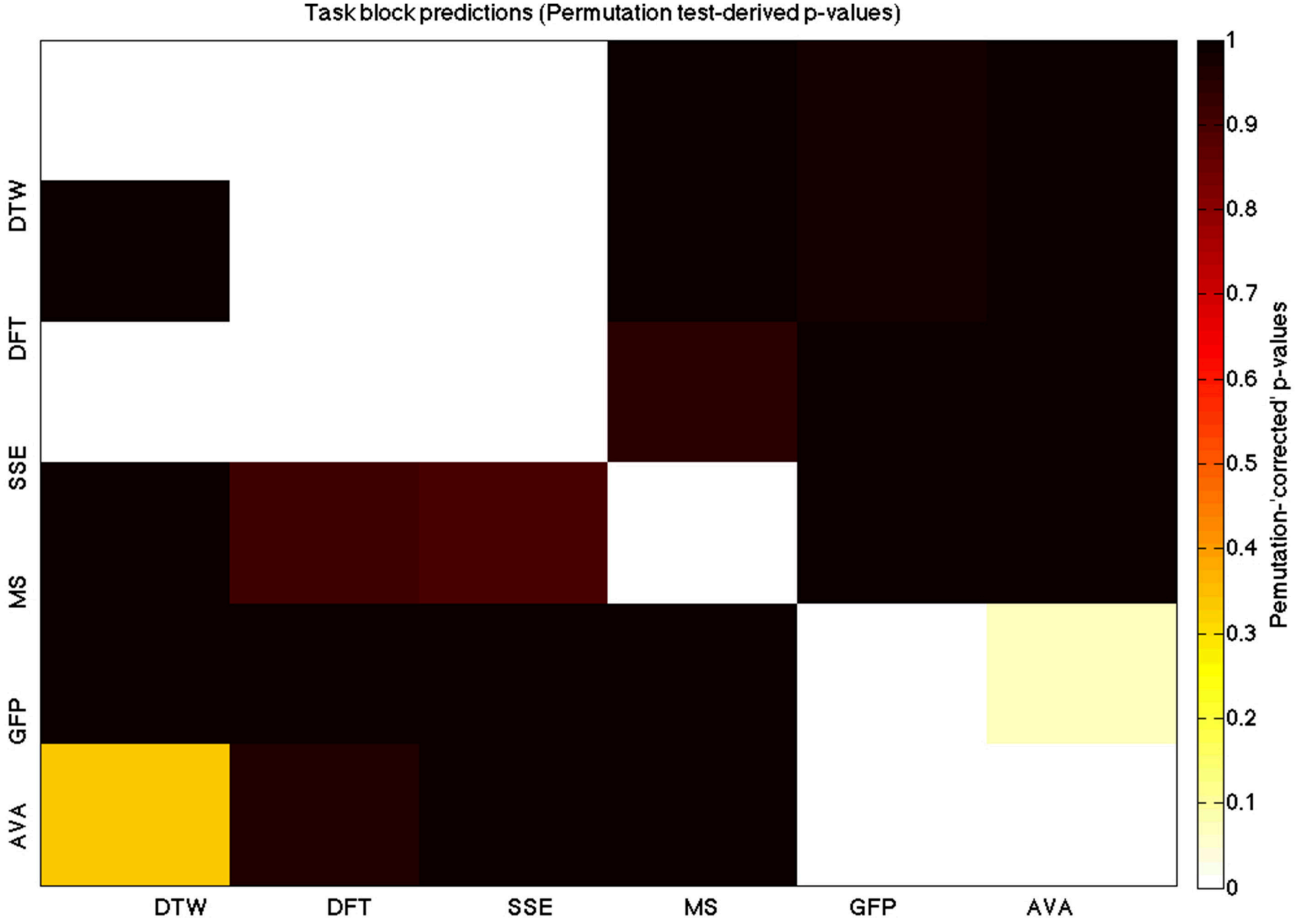
**A heatmap showing permutation tested group-level *p*-values from the *t*-tests on the GLM-derived beta coefficients of the task blocks**. The hotter the color (i.e., the closer to white) the closer the value is to 0. The diagonals are all set to 0, for clarity.

**Figure 7:**
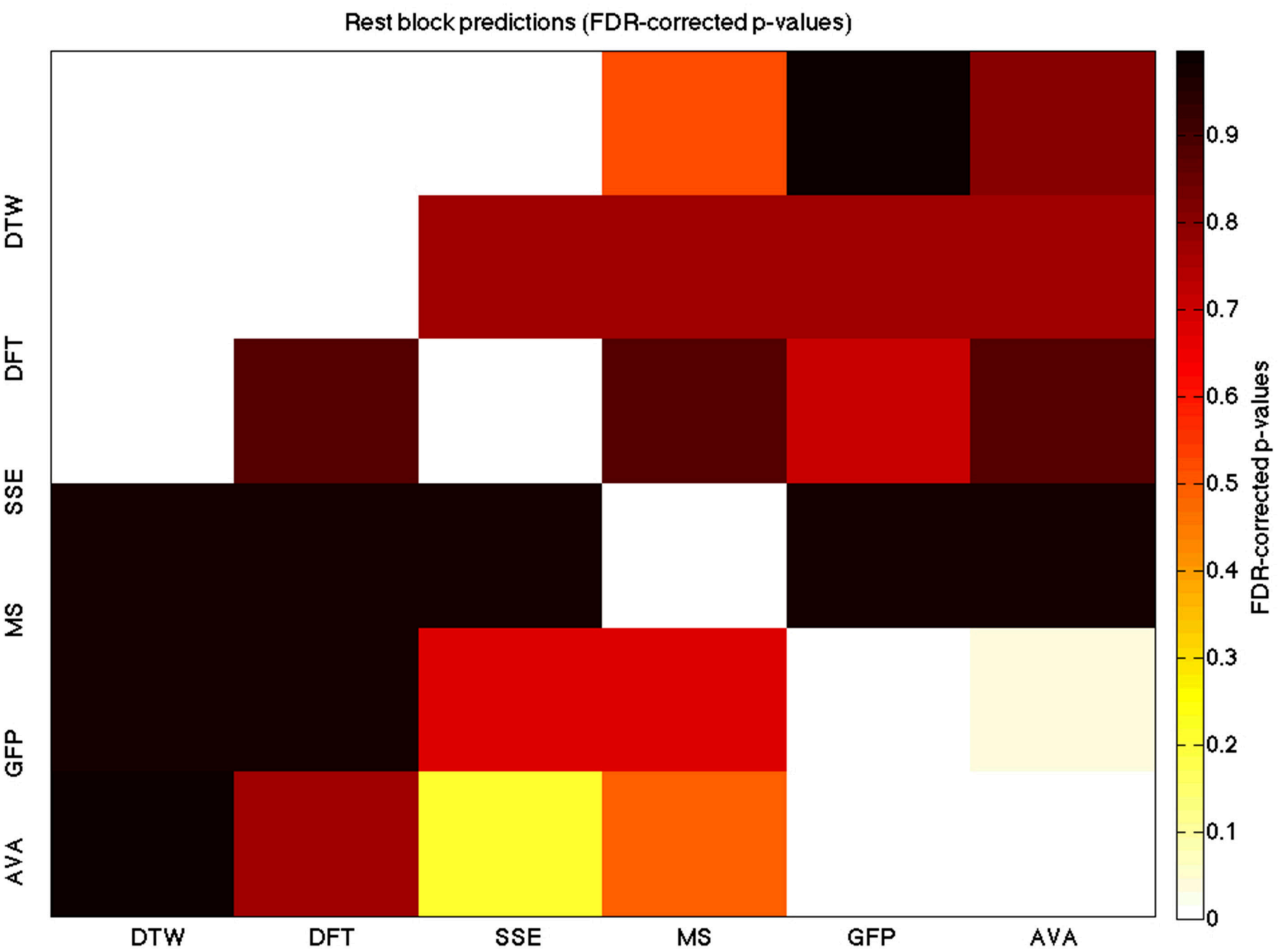
**A heatmap showing FDR-corrected group-level *p*-values from the *t*-tests on the GLM-derived beta coefficients of the rest blocks**. The hotter the color (i.e., the closer to white) the closer the value is to 0. The diagonals are all set to 0, for clarity.

**Figure 8:**
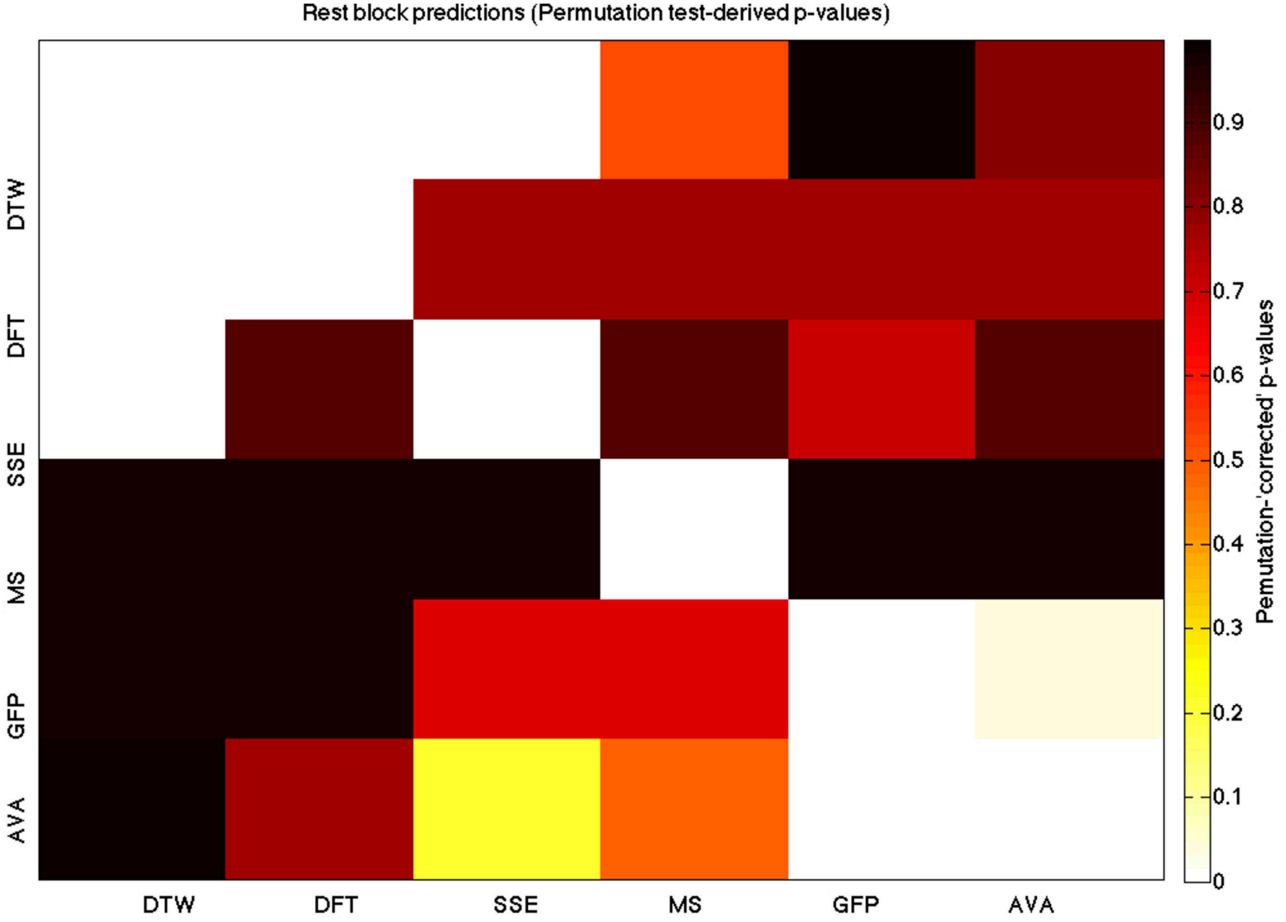
**A heatmap showing permutation tested group-level *p*-values from the *t*-tests on the GLM-derived beta coefficients of the task blocks**. The hotter the color (i.e., the closer to white) the closer the value is to 0. The diagonals are all set to 0, for clarity

We used a data set where we had alternating blocks of task periods and rest periods in order to be able to make a cognitively meaningful comparison and application of the methods here. As discussed previously, we wanted to see whether we could confirm and add additional evidence to the prevailing view that resting states are more variable and less predictable than task states. We present the results for task followed by rest group level results for each variable of interest. For each, we performed FDR-correction on *p*-values from a standard one-sample *t*-test on the GLM beta coefficients, as well as permutation testing on those post-GLM (and pre-FDR) *p*-values. We show both results in separate heat maps, for both task and rest. In Figures 5, 6 we show FDR-corrected and permutation-testing-derived *p*-values, respectively, for prediction on the task blocks. Figures 7, 8 show the FDR-corrected and permutation-testing-derived *p*-values, respectively, for prediction on the rest blocks during the task. The Figures are presented in the form of heat maps that show brighter/hotter colors for lower *p*-values. More detailed listing of *p*-values and results follows the heat map Figures, where in each case we first mention specifics of task, followed by specific results for the rest blocks. In each sub-section of specific results, we follow the ordering of the heat map variables—reporting results for DTW, DFT, SSE, MS, GFP, and AVA, in that order. All group-level results are FDR-corrected with alpha = 0.05. We also note that all *p*-values reported, unless otherwise stated, are either FDR-corrected or permutation-tested *p*-values on a group-level.

### DTW-spectrum vs. DFT-spectrum overview

We start by comparing in some more detail the DTW and DFT spectra, as these are (a) conceptually the most similar to each other and (b) DFT is the best understood and most-widely known from the methods we apply here. We remind here that the DTW spectrum here is a measure of how similar the EEG signal is to various sinusoids (as that is how the spectrum is computed). In Figure 6, we show a DFT spectrum and the corresponding DTW spectrum, for a range of frequencies between 8 and 13 Hz for one subject in three different trials (subject level data within a single model run). We observed this correlation between the DFT and DTW spectra in all subjects during both pre task and pre rest blocks, though resting blocks showed a (very slightly) weaker correlation.

**Figure 9:**
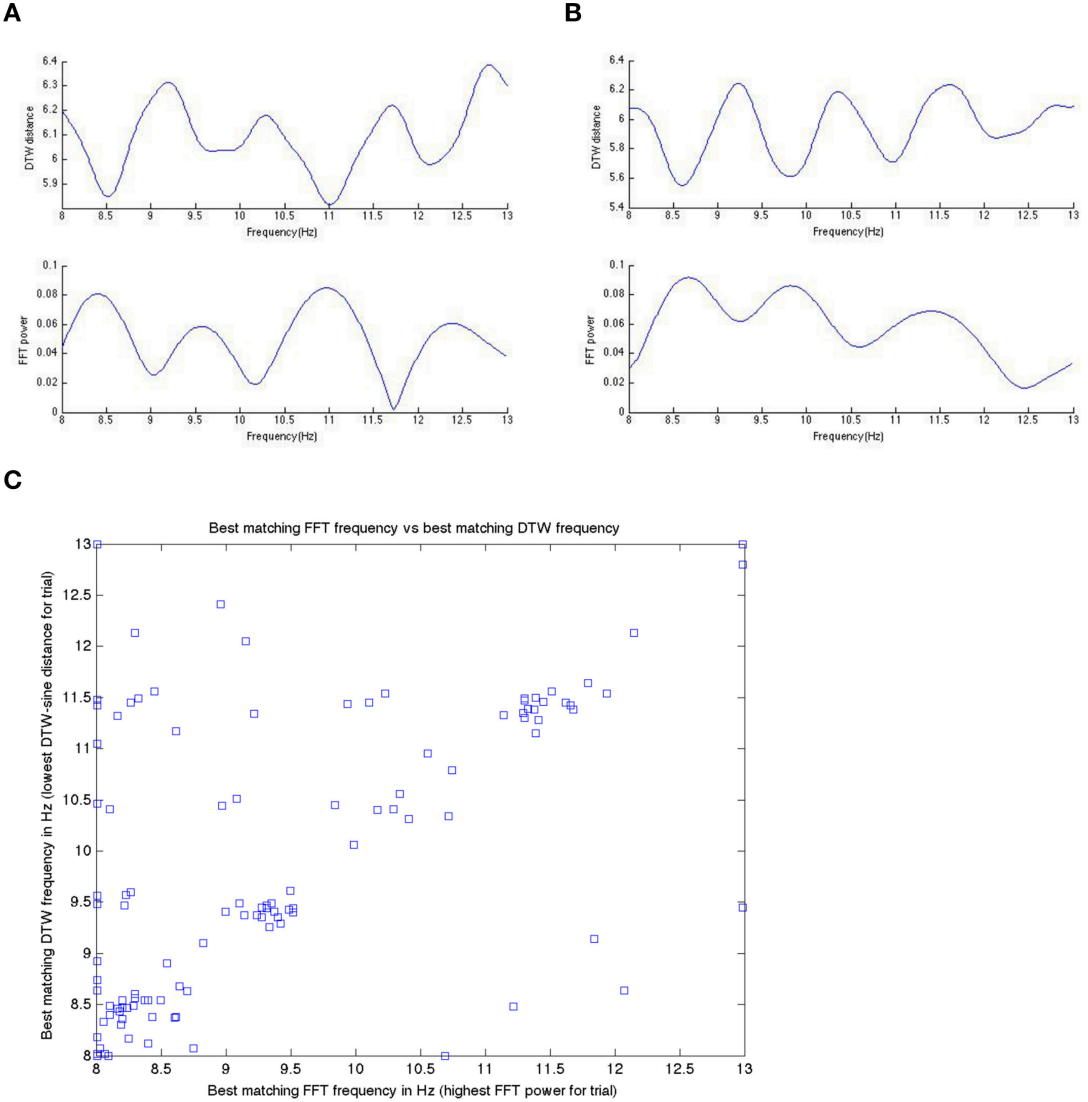
**In (A,B) here, we show the inverted nature of the DTW spectrum compared to the DFT spectrum for two different trials within a single subject**. We see the same pattern/behavior of the DTW vs. DFT in all trials in all subjects that we have looked at. Panels **(A,B)** show on the x-axis the frequency and on the y-axis the DTW distance or DFT power at that frequency bin. If we take the DTW-spectrum frequency bin with the lowest distance (best matching one) and the highest power DFT frequency bin (best matching one) and plot those for all trials, in one subject, we get **(C)**. Note that while the best matching frequencies nicely align along the diagonal (as they should if we get the same or nearly the same results with the DTW-spectrum and the DFT-spectrum), there are quite a few differences as well. We suggest that these differences are likely meaningful and useable in prediction, as we have done in this work.

Figure 9 shows a specific subject's comparison between DFT-spectrum and DTW-spectrum. It shows that one is roughly the inverted version of the other, but the two are not identical.

### DTW GLM results

As expected, on a group-level, for the task condition, we find that the DTW is best predicted by the DFT (FDR-corrected *p* < 10^−10^ and permutation *p* = 0.0002), though the SSE contributes additional explanatory power (FDR *p* < 0.0003 and permutation *p* = 0.0006). We note that the permutation testing suggests that the variance in DTW explained by the DFT and SSE may actually be of similar importance.

For the rest predictions, we find a similar pattern, with the DFT contributing possibly marginally more toward explaining the variability in the DTW (FDR *p* < 10^−13^ and permutation *p* = 0.0002) and (*p* < 10^−6^, respectively), than the SSE (FDR *p* < 10^−6^ and permutation *p* = 0.0002).

We note here that the DTW seems predictable in no small part also by the SSE, as opposed to our original expectation that the DFT would be sufficient (as they are highly inversely correlated). Since the GLM is taking into account all variables at the same time, this implies that both variables are adding useful model-explained variance—and indeed, similar amounts, toward modeling the DTW variance.

### DFT GLM results

For the task block predictions here, we find the same pattern as above, with the DTW and DFT. In other words, measure most predictive of the DFT is the DTW (FDR *p* < 10^−10^ and permutation *p* ~ 0), though the SSE seems to explain some additional variance not accounted for by the DTW variable (FDR *p* < 0.009 and permutation *p* = 0.0092).

For the rest block predictions, we find that the only variable predictive of the DFT is the DTW (*p* < 10^−11^ for FDR and *p* ~ 0 for the permutation test results).

We point out the main result here is not so much that the DTW is predictive of the DFT (which it is), but that the DTW is more predictive of the DFT (lower *p*-values from GLM) than the DFT is of the DTW. We also note that the task predictors are stronger than the rest predictors. We find both effects to be mostly consistent across repeated 20-model-averagings that we performed to validate these results.

### SSE GLM results

For task block predictions, we find that both the DTW and the DFT are the sole predictors of SSE (*p* = 0.0011 and *p* = 0.0013 for the FDR and *p* = 0.0004 and *p* = 0.0006 for the permutation test results). Note that the DTW is slightly more predictive than the DFT.

For the rest block predictions, we find that the only variable predictive of SSE is DTW (*p* < 10^−8^ for FDR and *p* ~ 0 for the permutation test results).

We find that the DTW outperforms the DFT in predicting SSE, in both task and rest conditions. Also, we find a higher predictability (across all variables) in the task blocks than in the rest blocks of the task.

### Microstate length GLM results

For task predictions, we find that the only weak predictor of the microstate length of the trial is the mean GFP power (FDR-corrected *p* < 0.05 and permutation *p* = 0.0646).

In the rest predictions, we find a marginally stronger prediction of the GFP to the microstate length (FDR *p* = 0.033 and permutation *p* = 0.0468).

It is almost certain that this difference in predictability between rest and task here is due to noise and randomness, rather than a real effect between task and block states, as we found variation by running multiple 20-run averages and we found at times the task predictability to be higher (i.e., lower *p*-values for the task case).

### Mean global field power GLM results

Interestingly, we find a much stronger effect in the other direction, with the microstate length being a significantly powerful predictor of the mean GFP power (FDR *p* = 0.00028 and permutation *p* = 0.0002), in the task condition.

In the rest condition, we find a similarly more powerful effect in this direction between the mean GFP and the microstate length (FDR *p* = 0.0052 and permutation *p* = 0.007).

In this instance, we found a regular higher predictability (lower *p*-values) in the task condition than in the rest condition, also in other 20-run averages that we looked at.

### Avalanche length GLM results

We report no significant and consistent predictors of the avalanche length immediately prior to the trial from any of the measures we used here, but some runs, and on some subjects, we found significant effects of microstate length on avalanche length, and vice versa. This effect could make biological sense and is potentially interesting, but we do not discuss it further here as it was not consistently observed and in any case not on the 20-run average results that we report here.

### Bold GLM results

There were very few to no consistently strong effects that remain on a group-level after averaging results from the 20 runs, as presented here. There were stronger individual effects or group effects with fewer averaging, however these were not always consistent across validation repetitions of the 20-run averaging that we discuss here. Any consistent (but weak) results may or may not have potential significance.

Nevertheless, for completeness, we summarize all potentially useful and interesting results that we found. The most consistent effects tended to be the microstate length or mean GFP. For example, in the 20-run average results we are reporting and discussing here, for the RSN14 GLM (which Smith et al., 2009) claim is biologically plausible as a fairly deep thalamus/caudate region (but may also be artifactual due to blood vessels), the mean GFP power has an FDR *p* = 0.06322 and permutation *p* = 0.0698 in the task condition.

Though we generally find higher predictability (especially in the EEG data) in task than in rest blocks, we find the opposite here. This is probably due to the nature of the resting state networks extracted from the BOLD data. Because these were taken during and apply especially to RSNs, they are more likely to be expressed during the resting blocks, as we find. We find few consistently very strong effects, but there are multiple weak but consistent effects that we've found. For the RSN12 GLM we found DTW to be slightly explanatory of the variance of the RSN (FDR *p* = 0.0996, permutation *p* = 0.141), RSN17 has the avalanche length as the strongest and only noticeable regressor (FDR *p* = 0.02055, permutation *p* = 0.032). RSN12 is not identifiable to a specific functional network but may be a combination of multiple biologically plausible functional networks (Smith et al., 2009) but RSN17 is of clearly blood vessel-related artifactual origin.

## Discussion

DTW is a powerful, flexible domain-general method for comparing sequences that has considerable potential for better characterizing neural signals. The purpose of this study was to see whether we could use DTW in novel ways to study brain dynamics, measured with EEG and FMRI. We reanalyzed an existing simultaneous and combined EEG-fMRI dataset (Fagerholm et al., 2015) to explore how useful DTW is at predicting a range of measures describing neural dynamics and how they are affected by cognitive state: including standard DFT approaches, the predictability of the EEG signal based on neural networks, EEG microstates, point-process neuronal avalanches, simultaneously acquired BOLD signal. We showed that DTW is generally the best predictor of other measures than any other (with the exception of avalanche length and microstates which weakly predicted each other in some cases). The DTW was also useful at comparing rest with active cognitive task states, where (as we predicted based on Fagerholm et al., 2015) DTW was a better predictor during task than rest, though other predictors displayed the same pattern of higher task predictability than rest predictability.

The DTW-spectrum resulted in a spectrum highly correlated to a standard DFT-computed spectrum but it also demonstrated additional variability in the data not accounted for by the DFT. This suggests that DTW may be a more useful and a more interpretable spectrum in the sense of how DFT is typically used—i.e., showing how much of a given frequency there is in a signal. The DTW-spectrum is more interpretable directly in this sense, compared to the DFT signal. The DTW measure seemed to more consistently and more strongly predict other variables.

We also add evidence to and confirm our initial general hypothesis that task states are more predictable and predictive contrasted to resting states using DTW measures. Though some variables of interest (like the DTW-spectrum and DFT-spectrum) are mutually predictive of each other strongly in both rest and task conditions, there are stronger effects in the task condition.

We also noted an interesting effect that we did not specifically expect or look for, correlating the trial/pre-trial microstate length and the trial/pre-trial mean GFP. In particular, we noted that the microstate length consistently and strongly predicted the mean GFP. Because this was not the focus of the study, we only suggest in passing that this could be because the longer the microstate is, the more likely it is to shift to another microstate, as microstates do not tend to persist for more than about 100 ms on average. Perhaps the longer the brain is in a certain global state (characterized by a given microstate), the more it attempts to shift to another microstate or global state, characterized by increased GFP and changing topography.

It is well recognized that noise has a substantial effect on MLP model training and may have contributed to some of the spurious associations. On the other hand, most of the strong associations are so far beyond chance (e.g., *p* < 10^−10^) that this is certainly not causing all associations observed. Therefore, having a cleaner dataset (not acquired simultaneously with FMRI) would help in decreasing the likelihood of noise driving any association. We would like to repeat and re-run this modeling and analysis pipeline on cleaner EEG-fMRI data sets as well as explore the use of the DTW-spectrum and other DTW-based techniques on other types of data sets and problems as well, as the DTW-spectrum approach is likely to prove useful beyond the uses explored here.

We conclude by remarking that DTW is an underexplored method for neuroscientific investigations which can be flexibly used not only to assess sequence similarity (and e.g., subsequent clustering of those sequences), as originally developed but also to aid characterizing the frequency spectrum of neural signals. We speculate that this marginally but significantly higher predictive power of the DTW-spectrum measure may be due to its ability to capture more oscillatory/sinusoidal dynamics compared to a DFT-type typical spectrum. Whether the differences between the DTW-spectrum and the corresponding DFT spectrum are indeed differences of oscillation vs. non-oscillation dynamics differentially captured by the two methods remains an open question, but one worth investigating further, as an affirmative answer here would suggest that the method may be highly applicable to the study of all sorts of oscillatory systems.

## Author contributions

MD and RLeech designed research; MD performed research; MD, RL, GS, EF, DS contributed unpublished analytic tools or the data; MD and RLeech analyzed data; MD, RLeech, and RL wrote the paper.

### Conflict of interest statement

The authors declare that the research was conducted in the absence of any commercial or financial relationships that could be construed as a potential conflict of interest.
